# Clinical Interdisciplinary Collaboration Models and Frameworks From Similarities to Differences: A Systematic Review

**DOI:** 10.5539/gjhs.v7n6p170

**Published:** 2015-04-15

**Authors:** Mousa Mahdizadeh, Abbas Heydari, Hossien Karimi Moonaghi

**Affiliations:** 1 Evidence-Based Caring Research Center, Department of Medical-Surgical Nursing, School of Nursing & Midwifery, Mashhad University of Medical Sciences, Mashhad, Iran; 2 Department of Medical-Surgical Nursing, School of Nursing and Midwifery, Mashhad University of Medical Sciences, Mashhad, Iran

**Keywords:** nurse-physician relations, care team, collaboration, interdisciplinary relations

## Abstract

**Introduction:**

So far, various models of interdisciplinary collaboration in clinical nursing have been presented, however, yet a comprehensive model is not available. The purpose of this study is to review the evidences that had presented model or framework with qualitative approach about interdisciplinary collaboration in clinical nursing.

**Methods:**

All the articles and theses published from 1990 to 10 June 2014 which in both English and Persian models or frameworks of clinicians had presented model or framework of clinical collaboration were searched using databases of Proquest, Scopus, pub Med, Science Direct, and Iranian databases of Sid, Magiran, and Iranmedex. In this review, for published articles and theses, keywords according with MESH such as nurse-physician relations, care team, collaboration, interdisciplinary relations and their Persian equivalents were used.

**Results:**

In this study contexts, processes and outcomes of interdisciplinary collaboration as findings were extracted. One of the major components affecting on collaboration that most of the models had emphasized was background of collaboration. Most of studies suggested that the outcome of collaboration were improved care, doctors and nurses’ satisfaction, controlling costs, reducing clinical errors and patient’s safety.

**Conclusion:**

Models and frameworks had different structures, backgrounds, and conditions, but the outcomes were similar. Organizational structure, culture and social factors are important aspects of clinical collaboration. So it is necessary to improve the quality and effectiveness of clinical collaboration these factors to be considered.

## 1. Introduction

Care is a team effort that its continuity is not possible by one person alone (Hall, Weaver, Gravelle & Thibault, 2007). Coordination, communication and working together are crucial for effective care. Interdisciplinary collaboration is defined as a complex phenomenon that is often formed between two or more people from various professional fields to achieve common goals (Houldin, Naylor, & Haller, 2004).

Nowadays, it is required that physician, nursing and other health-related professions in interdisciplinary context provide integrated care in a way that they must be avoided from separation (McCallin, 2001). However, now, the atmosphere of the clinical environment is not satisfactory and in most studies, it is reported that the conditions of interdisciplinary collaboration is poor (Fewster-Thuente, 2011). The best way of relation between these variables is explained in a model. Model is summarized showing of a complex theory and special events, structures and systems (Shearer, 2009).

Model is a set of general and abstract concepts that connect together and refers to interested central phenomenon of a discipline (Fawcett & Desanto-Madeya, 2012). Nursing models or frameworks create a conceptual vision and a guide for nursing practice (Alligood, 2006). So far, different interdisciplinary collaborations have been presented according to the clinical contexts.

Despite there are the different models of collaboration a limited number of models are available for scholars who wish to study interprofessional collaboration (Gaboury, Bujold, Boon & Moher, 2009). Development of models is often done with qualitative approach. The phenomenon of collaboration is a social process and its different dimensions were considered in the qualitative studies.

Therefore, this study was performed with the aim to review the evidences that had presented a model or framework with qualitative approach in interdisciplinary collaboration of clinical nursing. With studying of processes, backgrounds, outcomes, similarities and differences will be obtained comprehensive understanding of this social phenomenon.

## 2. Materials and Methods

In this study, all qualitative studies which had presented a model or framework in the field of clinical interdisciplinary collaboration between nurses and other disciplinary were evaluated. This study was performed based on the framework and data extraction form that was designed based on the objective of the study and inclusion criteria by the researchers. Data was extracted for each paper by two of the authors independently to enable them to independently judge the quality. This form included information of research methodology (objective, methodology, sampling, sample size, location, study type, and data collection tools).

The research questions were: 1. what are the backgrounds and consequences of models and frameworks for interdisciplinary collaboration in clinical nursing? 2. What are the similarities and differences between the model and frameworks of interdisciplinary collaboration of clinical nursing?

In this review, all the articles and theses published from 1990 to 10 June 2014 in both English and Persian which had performed interdisciplinary collaboration processes in the clinical nursing with qualitative approach and had presented a specific model or framework for nurse’s collaboration with other disciplines were studied. In this systematic review, an extend research was performed to find articles from English databases: Proquest, Scopus, pub Med, Science Direct, and Iranian databases of Sid, Magiran, Iran Mede, and interdisciplinary relations, nurse-physician relations, care team, collaboration, and their Persian equivalents were used as the key words, according with MESH. At the first, separated key words and then combination of key words were used by using AND/OR for combining the words. For example search term was used nurse-physician relation or nurses-doctors/medicine relation or that nurse and physician collaboration, care team or care group. To not miss the studies, review of the references of relevant studies was independently controlled.

Inclusion criteria included: (1) the studies that have at least a qualitative approach. 2. Articles and theses which had presented the model or framework for clinical collaboration. 3. Participation of clinical nurses. 4. The full text of articles or theses, 5. Assessment of original studies, and exclusion criteria included: (1) The models and frameworks that were not related to clinical nursing, 2- Inaccessibility to some of full texts.

Participants were nurses and other disciplines including doctors, social workers, physiotherapists, psychiatrists, psychologists that performed care and treatment of patients as a clinical discipline. In this review, all abstracts of relevant papers and theses and references were reviewed independently by the researchers and all those who had presented the model or framework for nursing collaboration were selected. In the next step, the researchers selected all full-text studies that had provided a model or framework for collaboration with independent review, and if there is a difference in a particular case, the discussion began.

Then, all the studies selected by the researchers were evaluated using the framework and the form designed for data extraction based on the objective of the study. For data extraction and synthesis, articles had been carefully read by one of the reviewers; the most important points of the articles were summarized along with the aim of the research and these points were extracted and sorted by narrative summary. Finally, findings were reported based on the research’s questions. For increasing the study accuracy, extracted data were controlled and reviewed through reviewing the process by other researcher. Evaluation was performed by some items of qualitative studies form (“Support Unit for Research Evidence (SURE),”).

**Figure 1:**
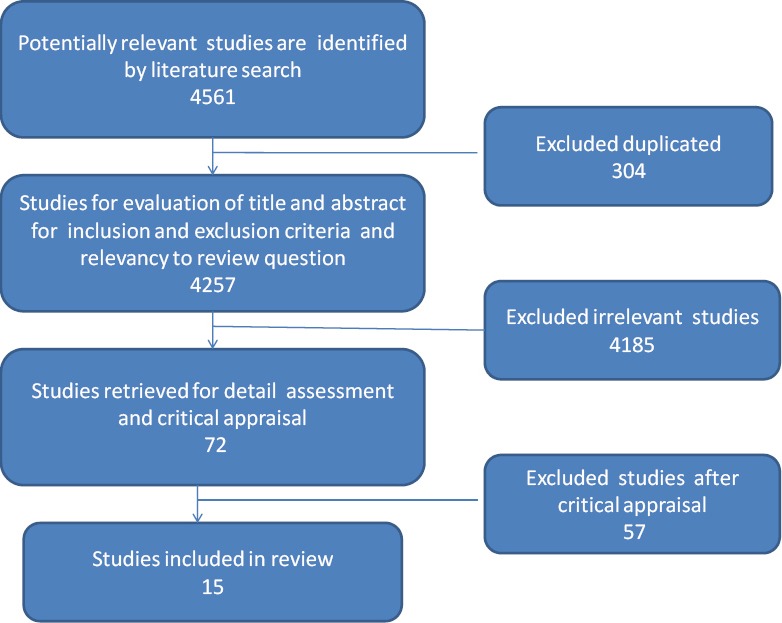
Process of searching for review

In this search, a total of 4561 articles and theses were found. Among these, 35 articles were in Persian found from internal databases. 3278 studies due to no participation of nurses in the process of collaboration and 1177 studies due to collaboration in other professional disciplines including research and education, also, 304 studies due to reprinting were excluded. Among 72 abstracts and theses, 57 cases which were performed with non-qualitative approaches or didn’t have collaboration framework or models were excluded and finally, 15 full texts including 13 full-text articles and 2 nursing PhD theses which had presented collaboration model in clinical nursing were evaluated (Figure 1). Most of the papers were published in international journals and databases; only one article was published in Iran.

## 3. Results

Extracted studies were conducted in clinical settings and were patient-centered and using this approach had presented a model or framework. In all studies, nurses were presented as one of the participants. Since nurses have most clinical collaboration and interaction with physicians, most studies had selected the physician as one of the other participants (Baggs et al., 1999; Collins, Bakken, Vawdrey, Coiera & Currie, 2011; Fewster-Thuente, 2011; Herrmann & Zabramski, 2005; Mueller et al., 2014).

In some studies, participants were from different disciplines such as psychologists, social workers, etc.(Douglas & Machin, 2004; fallahi kheshknab M, 2002; Gaboury et al., 2010; Légaré et al., 2011). In this review, from extracted papers and theses, 12 models (80%) and 3 frameworks (20%) were obtained. The research was performed with different qualitative approaches and methodology. Thus, sampling method, sample size, and the tools used in these studies were different

5 studies (33%) used grounded theory methodology, 3 studies (20%) used focus group, and 3 studies (20%) used an integrated approach. Also, 2 studies (13%) had used a phenomenological approach. Ethnography and action research also had assigned one case each one (6.6%). Canada country with 6 studies (40%) had devoted the highest number of publications. American country with 4 studies (26.6%) was the next rank. 3 studies (20%) were obtained from England, and Iran and Germany each one had published one study (6.6%) (Table 1).

**Table 1:** Characteristics of the review studies

Concepts	Objectives	Design/ sample size/ tools	Model / framework.	Author / Year / Country
The concepts of model were tools, facilitators and barriers	Providing a model for recording exchange of electronic interdisciplinary information in ICU	observation/5 interview,1 focus group/field note	Model	Collins& etal/2010/USA

Concepts were availability, location, time, knowledge, acceptance, respect, trust, interest, and questioning	Understanding the process of collaboration between physicians and nurses	Grounded theory/20/interview	Model	Baggs&Schmitt/1997/USA

The central concept were working together, moving towards a common goal, and several major categories, such as getting together and exchanging information	Understanding the process of collaboration between doctors and nurses and presenting theory	Grounded theory/22/interview	Model	Fewster/2011/USA

In this model, the main concepts were common vision and goals, client-centered, integration, formulating, government	Accreditation of indexes of collaboration model-providing typology of collaboration	Mixed /33/interview	Model	Damour/2008/Canada

In this clinical model, central concepts were physicians, nurses, patients,	Explain the benefits of the clinical collaboration between physicians and nurses	/18/interview	Model	Herrmann&Zambramki/2005/USA

Concepts of protection, power, field, group life in the form of classes were discussed	Improving the groups, success by using the theory of momentum project concept	Grounded theory/20/interview	Model	Douglass&machin/2004/UK

Various presented models were parallel collaboration, consultation, coordination, multidisciplinary, interdisciplinary, and consolidated	Providing thought framework to compare and evaluate different models of team-centered care function	Grounded theory /Workshop/ unclear	Framework	Boon&etal/2004/Canada

Concepts such as: patient’s environment, patient’s family, inter-professional team were the elements of decision model	Development of thought model with inter-professional approach for decision	mixed/Focus group/ interview	Model	Legare&etal/2010/Canada

The discussed concepts were education, trust, credibility, emotional burden	Describing the preparation and development of model of nurse-led follow-up care	phenomenology/12/interview	Model	Sally moore/2006/UK

Framework structures included the structure (confidence), process (knowledge exchange, physician-centered, collaboration conflicts) outcomes (satisfaction, personal growth, hope to life)	Discovery and explanation of the health care experiences and facilitating factors and limiting collaboration	mixed/qullity21/ 87quantity	Model	Gaboury &etal/2010/Canada

The characteristics of comprehensive model have been discussed in the form of these concepts: client, family, social skills, self-care, social and spiritual aspects	Designing of care model in multi-dimensional Psychological rehabilitation of patients with schizophrenia	Grounded theory/15/interview	Model	Fallah&etal/2002/Iran

Concepts were emphasized in three dimensions of data, process, and outcomes	Discovery and interpretation of experience of persons working in Canadian health care clinics	Grounded theory/21/interview	Framework	Gaboury &etal/2009/Canada

The concepts of collaboration process, factors affecting collaboration, the degree of collaboration in the framework of hypotheses were tested	Test of model and hypothesis	Qualitative/Questioner	Model	Hee lim/2008/south Korea

Concepts of this model were inter-professional collaboration, communication, interaction	Discovery of current situation of collaboration and relation between general practitioners and nurses of nursing homes and presenting model	mixed /observation/interview	Model	Mueller &etal/2014/Germany

Trainer, skilled practitioner, researcher, director of progress, consulter, colleague	Providing clinical and counseling model for nurses	Action research/ focus group/ interview /field note	Framework	Manley/1997/UK

In this review, 7 published studies (46.6%) had selected health care centers as research environment (Boon, Verhoef, O’Hara, & Findlay, 2004; D’Amour, Goulet, Labadie, Martín-Rodriguez, & Pineault, 2008; Gaboury et al., 2010; Légaré et al., 2011). Also, some studies were performed in hospitals (Baggs et al., 1999; Collins et al., 2011; Moore et al., 2006). In most studies, the main structure was three components of collaboration process, context of collaboration and consequences that they were emphasized.

### 3.1 Backgrounds of Collaboration

One of the major components affecting on collaboration that most of the models had emphasized was background of collaboration. Focus of background was on the roles of people, management, the external environment, and atmosphere regnant on the group (4). Organizational structure was important concept that many studies had discussed as the background of collaboration and the affecting conditions (Baggs et al., 1999; Boon et al., 2004; D’Amour et al., 2008; Douglas & Machin, 2004; Gaboury et al., 2010).

In this regard, (D’Amour et al., 2008) in defining their model structure introduced four dimensions and ten indicators; two dimensions of these four dimensions of proposed model were related to the organization. These two dimensions included the formalization of the organization and it’s Governance. In the study of (Douglas & Machin, 2004), 6 categories were extracted that one of these categories was background. The researchers believed that background is defined as what affects the group condition such as the professional aspects, broad organizational and managerial aspects which affect the outcomes.

Collins et al. (2011) had pointed to environmental concepts, tools, facilitators and barriers as the background of their collaboration framework. Some studies had evaluated social and cultural factors as context variables (Fallahi Kheshknab M, 2002). In a study, the presented framework had three basic parts such as field of wok such as beliefs, values, organizational authority and hierarchical management (Manley, 1997). Légaré et al. (2011) mentioned the environment as the general background including the social norms, organizational routines and organizational structure.

### 3.2 Outcomes of Collaboration

Most performed studies reported that the outcomes of interdisciplinary collaboration of clinical nursing were positive. (Fewster-Thuente, 2011) led to the theory of working together. Core category of this theory of work together was towards a common goal. 9 main categories had determined the formation process of collaboration. This theory can be used as a guide for nurses and doctors.

The consequences of this theory were improving care, doctors and nurses’ satisfaction and controlling costs and reducing clinical errors and patient’s safety. In another research, the model of collaboration between the physician and nurse was presented. This model has a core variable of working together that the process of working was formed based on it. This concept has a set of antecedents such as: availability (place, time, knowledge) and acceptance (interest, discussion, active listener, openness, questioning, respect and trust). Model’s outcomes were the improved quality of care, satisfaction, better learning and controlling costs (Baggs et al., 1999).

Some other studies also emphasized on patient and nurse’s satisfaction as the outcome of the study (Fewster-Thuente, 2011; Gaboury et al., 2010; Herrmann & Zabramski, 2005). Cost effectiveness was an important factor that some researchers had proposed (Boon et al., 2004; Fewster-Thuente, 2011). One of the concepts that some authors have pointed out was improving the quality of care (Boon et al., 2004; Moore et al., 2006). In a multi-dimensional designed model, model’s center was the client and family of the client (Fallahi Kheshknab M, 2002).

Some studies had proposed that their model’s indicator is patient-centered (Collins et al., 2011; D’Amour et al., 2008). In another study, one of the concepts of presented model was care continuity (Moore et al., 2006). In the study by (Manley, 1997), the final outcome of collaboration framework was the quality of services provided for the patients. Reduced length of hospital stay was another consequence of the studies (Herrmann & Zabramski, 2005). Patient’s safety was another variable that some studies had pointed out (Collins et al., 2011). Exchange of knowledge and information also were the results of some authors (D’Amour et al., 2008; Fewster-Thuente, 2011; Gaboury et al., 2010).

### 3.3 Process

Focus of all studies was on the process of clinical interdisciplinary collaboration. Most researchers determine effective collaboration elements and indicators of the effectiveness of group work to understand the process of collaboration (Lim, 2008). (Fewster-Thuente, 2011) suggested that the process of collaboration was based on performance and believed that this process cannot be determined with review studies and should be performed by experimental studies in the presence of the participants.

The main question in the study of (Baggs et al., 1999) was about the interaction between doctors and nurses. In this study, the concept of working together was discussed as the core of process. In the model of (Légaré et al., 2011) also the process of collaboration in organization was emphasized. In the model proposed by (Boon et al., 2004), four key elements were discussed in the development of the model. These elements included the philosophy, values, structure and process.

In a study, the process of collaboration had been discussed in 3 dimensions including 1-the process of increasing communication, 2- reducing the independence of practitioners, 3-respecting to the opposed ideas and important decisions based on the results (Gaboury et al., 2010). In the model of inter-professional collaboration, the process of collaboration included concepts of communication, barriers, patient’s referral, and power of relations (Gaboury et al., 2009).

In a study, three variables of time constraints, lack of resources and the imbalance of power between health professionals were introduced as the barriers of collaboration and education, motivation for obtaining an inter-professional approach and mutual understanding of interdisciplinary roles were as the facilitator (Légaré et al., 2011). (D’Amour et al., 2008) had discussed high level of management as internal and external factors in the promotion of collaboration.

In another study, two hypotheses were evaluated which were related to the process of collaboration. The first hypothesis was that the characteristics of team members and context variables have a direct effect on the process of collaboration. The second hypothesis was that the characteristics of team members and context variables have a direct effect on the degree of collaboration. In this study, both two hypotheses were rejected, but the model was edited and modified after the test (Lim, 2008). These studies were different in some variables realated to qualitative researches (Table 2).

**Table 2:** The form of critical appraisal of qualitative studies

QUESTIONS FOR EVALUATION	Yes	Can’t tell	No
1- Does the study address a clearly focused question/hypothesis	All except boon et al, Herrmann et al.	Herrmann et al.	boon et al.
Setting?	All except boon et al.		boon et al.
Perspective?	All except boon et al, Herrmann et al, Fallah et al.	boon et al, Herrmann et al, Fallah et al.	

2- Is the choice of qualitative method appropriate? Is it an exploration of behaviour/reasoning/ beliefs)? Do the authors discuss how they decided which method to use?	All except boon et al, Herrmann et al, Collins et al, Guboury et al (2010).	boon et al, Herrmann et al, Collins et al, Guboury et al (2010).	

3- Is the sampling strategy clearly described and justified? Is it clear how participants were selected? Do the authors explain why they selected these particular participants? Is detailed information provided about participant characteristics and about those who chose not to participate?	All except boon et al, Herrmann et al, Guboury et al (2010).	boon et al, Herrmann et al, Guboury et al (2010).	

4-Is the method of data collection well described? Was the setting appropriate for data collection? Is it clear what methods were used to collect data? Type of method (focus groups, interviews, open questionnaire etc) and tools	All except boon et al.	boon et al.	

5-Is the relationship between the researcher(s) and participants explored?	All except boon et al, Herrmann et al, Mueller et al..	Herrmann et al.	boon et al, Mueller et al.

6- Are ethical issues explicitly discussed? Is there sufficient information on how the research was explained to participants? Was ethical approval sought?	Hee lim(2008), Fewster (2011).Gaboury (2009).	boon et al.	All except boon et al, Hee lim(2008), Fewster (2011).Gaboury (2009).

7- Is the data analysis/interpretation process described and justified? Is it clear how the themes and concepts were identified in the data?	All except boon et al, Collins et al, Douglas et al, Herrmann et al.	boon et al, Collins et al, Douglas et al.	Herrmann et al,

8- Are the findings credible? Are there sufficient data to support the findings?	All except Bagss et al, Sally Moore et al, Gaboury et al (2009) & (2010), Fallah et al, Herrmann et al, boon et al.	Bagss et al, Sally Moore et al, Gaboury et al (2009) & (2010), Fallah et al.	Herrmann et al, boon et al.

9- Is any sponsorship/conflict of interest reported?	boon et al, Mueller et al,	Fewester (2011), Hee lim (2008)	All except Fewester (2011), Hee lim (2008), Mueller et al, boon et al. Damour et al.

10- Finally…consider: Did the authors identify any limitations? Are the conclusions the same in the abstract and the full text?	All except Bagss et al, boon et al, Fallah et al, Manley et al, Douglas et al, Herrmann et al.		Bagss et al, boon et al, Fallah et al, Manley et al, Douglas et al, Herrmann et al.

## 4. Discussion

### 4.1 The Process of Collaborations

All studies claimed that they presented a model or framework that can be used in practice. Since these studies used a qualitative approach and methodology, had considered their studies’ limitations in terms of transformability and function (Collins et al., 2011; D’Amour et al., 2008; Fewster-Thuente, 2011; Lim, 2008). In all these models and frameworks, nurses participated as the main members of health care team who are permanently present in clinical situations; they are considered as the main elements of the model and were discussed as a meta-paradigm in the models and frameworks.

In these studies, the structures were formed based on the social and symbolic interactions and most of them discussed the phenomenon of collaboration from social dimensions. In the structures, three components of process, backgrounds and consequences were discussed from various angles which we will refer to them.

In the study by (Fewster-Thuente, 2011), working together towards a common goal was the base of the process of nurse and physician collaboration as a social process. The steps of this process are formed in the form of a set of categories such as paying attention to needs, knowing that with whom negotiations are done, finding honesty in a people, becoming one, the exchange of information and ideas, making plans, being in the same situation, making the situation, and monitoring the process.

In this study, we used an appropriate approach to evaluate the process of collaboration between nurses and physicians, but all the necessary tools such as observation and writing remembering were not mentioned. In the interview form, there were some questions that the ideas and attitudes of the participants were evaluated. For example, it was asked that what is your opinion about collaboration? Or whether you believe that collaboration should be done face to face.

In the study of (Baggs et al., 1999), the core of the collaboration process had three major concepts, the first was working together as a team. The second concept which is named as the heart of working together was patient-centered. The last extracted concept was participation partnership; these researchers had selected grounded approach to study the process of collaboration.

In this study, the tools of observation, field writing and memos were not used and only semi-structured interview was used for data collection. (Gaboury et al., 2010) had evaluated the collaboration process in 3 dimensions of knowledge exchange, the physician acceptance and managing participation among members of the team, conflicts related to inter-professional collaboration that it seems that except the first concept, the other ones are often in the form of contextual variables

In the study of (Lim, 2008) in addition to the process of collaboration, the degree of collaboration and factors affecting this process were also discussed. In this study, the researcher used the tools of questionnaire for evaluation of the process and the tools of interview and observation of area writing were not used, while the questions were less about the process. Since the studies of (Douglas & Machin, 2004; Manley, 1997) were action research, the tool of observation in this research was of particular importance, while these researchers had only used the interview as data collection and the processes were examined.

### 4.2 Backgrounds of Collaboration

In this study, the backgrounds were different. Some studies had correctly considered the backgrounds and conditions and antecedents in order to form concepts of paradigm and development of the model (Baggs et al., 1999; Fewster-Thuente, 2011; Lim, 2008). Boon et al. (2004) had pointed out the organizational structure as a background. Of course, the researchers only considered a small dimension of organizational dimensions including roles and hierarchy. D’Amour et al. (2008) with regard to the goal of their study reported that internal and inter-organizational areas were in a wider territory.

Légaré et al. (2011) had considered the environment as a total context included social norms, cultural values, organizational structure, and organizational routines. These concepts were in accordance with the concepts related to the area in the study of (Douglas & Machin, 2004) and (Manley, 1997) as well as (Gaboury et al., 2010) that evaluated the collaboration in the form of system input.

### 4.3 The Outcomes of Collaboration

What (Fewster-Thuente, 2011) had proposed as a consequence of collaboration between doctors and nurses was very close to the outcome of the study of (Baggs et al., 1999) These concepts were similar to the findings of the studies of (Gagliardi, Dobrow, & Wright, 2011; Patterson & McMurray, 2003). That was performed with different approaches. Thus, although these studies had different backgrounds and conditions, but were very similar in terms of the outcomes.

Since nursing is an international profession and the structure and professional relationships have functional patterns very close together, it can be assumed acceptable. Because health care providers with any conditions and structures are seeking for a common goal and they will use all the tools and capacity to achieve this important. In (fallahi kheshknab M, 2002) results client and family-oriented, teamwork, client self-sufficient, strong relationship of members, coordination with the roles of the nurse were the characteristics and the outcomes of multidimensional rehabilitation nursing model. Regarding the aim of this study, these concepts are considered unique outcomes of this study. This study had some limitations including: 1- the studies had models and frameworks, therefore, experimental studies were automatically excluded., 2. We didn’t have access to all theses specially those in Persian language, 3- The quality of some studies was not suitable to perform more complete review based on qualitative tools, 4. Studies were limited in English and Persian languages.

## 5. Conclusion

These studies had emphasized the importance of clinical collaboration between nurses and physicians. In all models and frameworks, nurses were considered as a main member of the care team and were discussed as a meta-paradigm. These studies had explained three components of the process, context and outcomes. Studies had different backgrounds and processes, but had the same consequences. Organizational structure, culture and social factors are important aspects of clinical collaboration. So it is necessary to improve the quality and effectiveness of interdisciplinary collaboration these factors to be considered. Most of the studies suggested that the outcome of collaboration were improved care, physicians and nurses’ satisfaction, controlling costs, reducing clinical errors and patient’s safety. If good clinical collaboration happens among physicians and nurses; patient, Patient’s family, nurses and physicians and organizations will benefit.

## References

[ref1] Alligood M. R, fallahi kheshknab M, m. S, shamlo S, abedi H.A, babae GH (2006). Introduction to nursing theory: its history, significance, and analysis. Nursing theorists and their work.

[ref2] Baggs J. G, Schmitt M. H, Mushlin A. I, Mitchell P. H, Eldredge D. H, Oakes D, Hutson A. D (1999). Association between nurse-physician collaboration and patient outcomes in three intensive care units. Critical care medicine.

[ref3] Boon H, Verhoef M, O’Hara D, Findlay B (2004). From parallel practice to integrative health care: a conceptual framework. BMC Health Services Research.

[ref4] Collins S. A, Bakken S, Vawdrey D. K, Coiera E, Currie L (2011). Model development for EHR interdisciplinary information exchange of ICU common goals. International journal of medical informatics.

[ref5] D’Amour D, Goulet L, Labadie J.-F, Martín-Rodriguez L. S, Pineault R (2008). A model and typology of collaboration between professionals in healthcare organizations. BMC Health Services Research.

[ref6] Douglas S, Machin T (2004). A model for setting up interdisciplinary collaborative working in groups: lessons from an experience of action learning. Journal of psychiatric and mental health nursing.

[ref7] Fawcett J, Desanto-Madeya S (2012). Contemporary nursing knowledge: Analysis and evaluation of nursing models and theories: FA Davis.

[ref8] Fewster-Thuente L (2011). Working together toward a common goal: A grounded theory of nurse-physician collaboration. LOYOLA UNIVERSITY CHICAGO.

[ref9] Gaboury I, Boon H, Verhoef M, Bujold M, Lapierre L. M, Moher D (2010). Practitioners’ validation of framework of team-oriented practice models in integrative health care: a mixed methods study. BMC Health Services Research.

[ref10] Gaboury I, Bujold M, Boon H, Moher D (2009). Interprofessional collaboration within Canadian integrative healthcare clinics: Key components. Social Science & Medicine.

[ref11] Gagliardi A. R, Dobrow M. J, Wright F. C (2011). How can we improve cancer care? A review of interprofessional collaboration models and their use in clinical management. Surgical oncology.

[ref12] Hall P, Weaver L, Gravelle D, Thibault H (2007). Developing collaborative person-centred practice: A pilot project on a palliative care unit. Journal of Interprofessional Care.

[ref13] Herrmann L. L, Zabramski J. M (2005). Tandem practice model: A model for physician - nurse practitioner collaboration in a specialty practice, neurosurgery. Journal of the American Academy of Nurse Practitioners.

[ref14] Houldin A. D, Naylor M. D, Haller D. G (2004). Physician-nurse collaboration in research in the 21st century. Journal of Clinical Oncology.

[ref15] Légaré F, Stacey D, Gagnon S, Dunn S, Pluye P, Frosch D, Graham I. D (2011). Validating a conceptual model for an inter-professional approach to shared decision making: a mixed methods study. Journal of evaluation in clinical practice.

[ref16] Lim K. H (2008). Collaboration between disciplinary teams caring for elders in Korean community settings: ProQuest.

[ref17] Manley K (1997). A conceptual framework for advanced practice: an action research project operationalizing an advanced practitioner/consultant nurse role. Journal of clinical nursing.

[ref18] McCallin A (2001). Interdisciplinary practice–a matter of teamwork: an integrated literature review. Journal of clinical nursing.

[ref19] Moore S, Wells M, Plant H, Fuller F, Wright M, Corner J (2006). Nurse specialist led follow-up in lung cancer: The experience of developing and delivering a new model of care. European Journal of Oncology Nursing.

[ref20] Mueller C. A, Tetzlaff B, Theile G, Fleischmann N, Cavazzini C, Geister C, Hummers Pradier E (2014). Interprofessional collaboration and communication in nursing homes: a qualitative exploration of problems in medical care for nursing home residents–study protocol. Journal of advanced nursing.

[ref21] Patterson E, McMurray A (2003). Collaborative practice between registered nurses and medical practitioners in Australian general practice: moving from rhetoric to reality. Australian Journal of Advanced Nursing.

[ref22] Pamela G, Reed Nelma B, Crawford Sh (2009). Perspectives on nursing theory.

